# The interdependence of substance use, satisfaction with life, and psychological distress: a dynamic structural equation model analysis

**DOI:** 10.3389/fpsyt.2024.1288551

**Published:** 2024-02-09

**Authors:** Fredrik D. Moe, Aleksander Erga, Jone Bjornestad, Ulrich Dettweiler

**Affiliations:** ^1^ Department of Mental Health, Haukeland University Hospital, Bergen, Norway; ^2^ Centre for the Study of the Sciences and the Humanities, University of Bergen, Bergen, Norway; ^3^ Department of Social Studies, Faculty of Social Sciences, University of Stavanger, Stavanger, Norway; ^4^ Centre for Alcohol and Drug Research, Stavanger University Hospital, Stavanger, Norway; ^5^ Norwegian Centre for Movement Disorders, Stavanger University Hospital, Stavanger, Norway; ^6^ TIPS – Network for Clinical Research in Psychosis, Stavanger University Hospital, Stavanger, Norway; ^7^ Department of Psychiatry, District General Hospital of Førde, Førde, Norway; ^8^ Cognitive and Behavioural Neuroscience Lab, University of Stavanger, Stavanger, Norway

**Keywords:** substance use disorder, recovery, longitudinal, dynamic structural equation model, polysubstance use

## Abstract

**Introduction:**

Longitudinal studies with annual follow-up including psychological and social variables in substance use disorder recovery are scarce. We investigated whether levels of substance use, satisfaction with life, and psychological distress fluctuate across five years in relation to having drug-free friends.

**Methods:**

A prospective naturalistic cohort study of change trajectories in a cohort of people diagnosed with substance use disorder and using multiple substances with quarterly and annual follow-up over five years. Two-hundred-and-eight patients were recruited from substance use disorder treatment in Rogaland, Norway. Out of these, 164 participants fulfilled the inclusion criteria. We used Bayesian two-level dynamic structural equation modelling. The variable ‘drug-free friends’ was assessed by a self-reporting questionnaire, while psychological distress was assessed using the Symptoms Checklist 90 Revised. Satisfaction with life was assessed using the Satisfaction With Life Scale while drug use was assessed using the Drug Use Disorders Identification Test.

**Results:**

The main findings are that higher-than-average psychological distress at a three-month lag credibly predicts higher-than-normal substance use at the concurrent time point t. Substance use and satisfaction with life seem to have synchronous trajectories over time, i.e. as the first decreases the latter increases and vice versa. During the five years after treatment, the participants mainly experienced a decrease in substance use and increase in satisfaction with life.

**Conclusion:**

Since the participants experienced positive and negative fluctuations for several years after treatment, it seems crucial to establish a dialogue with treatment professionals in order to create functional solutions for maintaining motivation and aiding recovery.

## 1 Introduction

Substance use disorder (SUD) research mainly consists of short-term investigations with substance use as the primary outcome measure (1, 2). Longitudinal studies with follow-up with a time scope extending two years (3), with repeated measuring points, are scarce. SUD is considered a long-term disorder (4), characterised by a cycle of abstinence and relapse (5). Thus, there is a need for longitudinal studies investigating the role of psychological and social functioning in treatment entry and after discharge.

The median time from first substance use to one year of abstinence is 27 years, while the median time from first treatment episode to one year of abstinence is nine years (6), indicating that achieving abstinence often takes several years. Moreover, the risk of relapse looms continuously. Two-thirds of patients relapse one year after SUD treatment (7), and there is still a risk of relapse after four to five years of continued abstinence (8).

Polysubstance dependence is common in clinical samples, although it is not a specified diagnosis in the DSM-5 (9, 10). In agreement with previous research on the STAYER sample (11), ‘polysubstance use disorder’ (PSUD) refers to problematic use of multiple substances where patients reported use of multiple substances within the last year of inclusion. People with PSUD are prone to more adverse effects on mental health compared to those with mono-substance use disorders (12, 13). Individuals suffering from PSUD may be more exposed to multicomorbidity, i.e. other mental and somatic disorders and other chronic illnesses (14).

Satisfaction with life is a key motivator and predictor of successful SUD treatment (15). Psychological distress is high among SUD patients (16). However, psychological distress is often reduced in conjunction with treatment entry or achieving abstinence, which may result from decrease in symptoms of withdrawal (17–19). Thus, reduction in withdrawal symptoms may moderate the strength of the relationship between treatment entry or achieving abstinence and psychological distress.

As relapse risk is present both in the initial and later phases of recovery, it may be valuable to investigate the dynamic and developmental change processes of SUD trajectories. Dynamic change processes refer to changes that occur more subtly than developmental ones. The focus is not on the overall trend across several years, e.g. measured annually, but on the dynamics of a fixed process (20), measured with autoregressive, i.e. time-lagged variables. Understanding SUD recovery as a dynamic and developmental change process makes it possible to assess whether factors affecting recovery, such as satisfaction with life, influence substance use levels differently in the early and late course of recovery.

In this study, based on a five-year follow-up SUD treatment sample, we will assess developmental change processes and explore differences between the participants in substance use as well as accounting for their individual change dynamics by assessing satisfaction with life, and psychological distress and if they are related to participants having drug-free friends. We hypothesize that having drug-free friends will be associated with reduction in substance use, increase in satisfaction with life, and decrease in psychological distress.

## 2 Materials and methods

### 2.1 Sample

We recruited the study sample (n=208) from the ongoing Norwegian Stavanger Study of Trajectories in Addiction (STAYER) - a prospective naturalistic cohort study of change trajectories among people diagnosed with SUD, investigating the course and timing of neurocognitive and psychosocial factors, including recovery (21, 22). Participants were recruited between March 2012 and December 2015, at the start of treatment in outpatient or residential treatment facilities in the Stavanger region of Norway. The sample consists of patients with SUD, alcohol dependence, and behavioural addictions. The STAYER study has been approved by the Regional Ethical Committee (REK 2011/1877). All participants provided written informed consent.

We included participants who were: 1) starting a new treatment sequence within addiction treatment services; 2) aged ≥16; 3) enrolled in a treatment programme to which they were admitted for at least two weeks; 4) categorised as having PSUD, i.e. had one substance use disorder diagnosis but reported use of multiple substances within the last year of inclusion. Of the 208 participants in the STAYER study, 164 met these criteria and were included. At enrolment, participants were between 16 and 51 years old (mean age: 27.1 years, with 7.1 years standard deviation); 62.2% were male; 93.7% of the cohort was born in Norway.

At baseline, N=164 participants provided data on their satisfaction with life, but only 146 participants on substance use and 109 on psychological distress.

Attrition during the five-year follow-up period was mainly attributed to withdrawal from the study (14%), death (5%), and lost to follow-up or other reasons (10%). This resulted in data being available for N=146 (89%) participants at one year (**Table 1**), N=133 (81%) at two years, N=121 (74%) at three years, and N=113 (69%) at four years. Details on the STAYER study methodology and retention are published elsewhere (22). All data were used for statistical analysis (cf. chapter “missing data”). **Supplementary Table 1** in the supplement gives an overview of the number of observations per variable at the respective measurement occasions.

### 2.2 Measures

*Drug use* was assessed using the Drug Use Disorders Identification Test (DUDIT) (23). The DUDIT has been found to have high reliability and validity (23, 24). We used DUDIT-C, which consists of the first two items of DUDIT, measuring the consumption of drugs (25). DUDIT-C was also used to make dichotomous variables used in the relapse calculation (detailed below). The cut-off for substance use was set at 0, comparing the participants scoring zero on DUDIT-C to all others. In the following “DUDIT-C” will be referred to as “DUDIT”.

*Drug-free friends* was assessed using a self-reporting questionnaire (KVARUS) to measure social support. ‘Drug-free friends’ has previously been used to measure social resources (26, 27). This variable was measured using a dichotomous question (YES/NO) at baseline and follow-ups: ‘Do you have friends without a history of substance use?’.

*Psychological distress* was assessed using the Symptoms Checklist 90 Revised (SCL-90-R), a 90-items self-report measure (28). The SCL-90-R has been found to have high validity and reliability (29). Items are scored on a five-point Likert scale ranging from 0 (not at all) to 4 (severe). The SCL-90-R consists of nine symptoms dimension subscales: Somatisation, Interpersonal Sensitivity, Obsessive-Compulsive Disorder, Anxiety, Depression, Phobic Anxiety, Hostility, Psychoticism, and Paranoid Ideation. Additionally, it includes a global severity index (GSI) and seven items that did not fit in any of the nine categories. The SCL-90-R has previously been used on this sample to measure psychological distress (11). In this study we used raw scores from the SCL-90-R GSI, which is comprised of the mean of all the items in SCL-90-R. The GSI is the most commonly used index from SCL-90-R.

**Figure 1:**
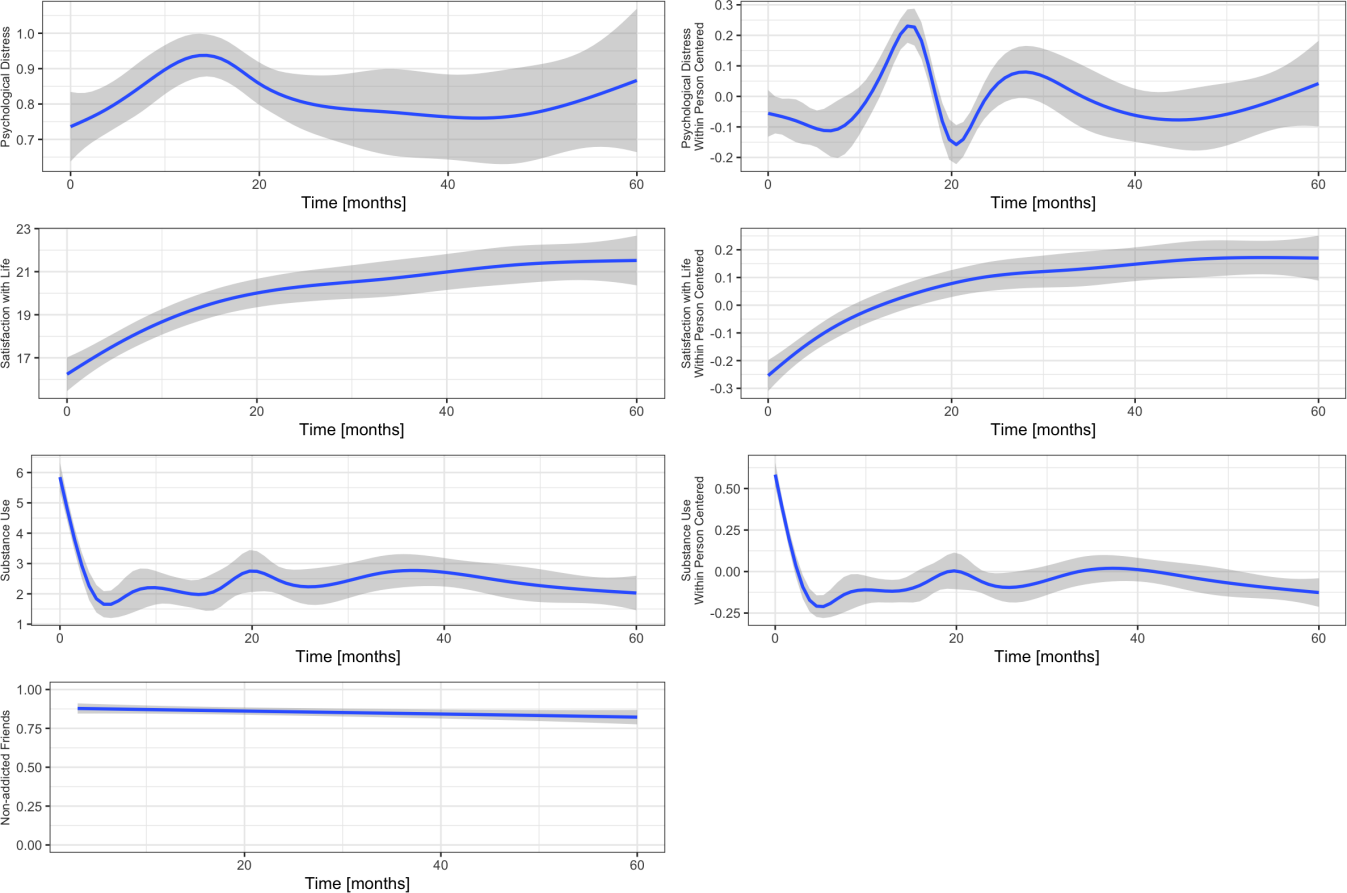
Display of the study variables with regression splines to indicate their trajectories over time. The left panel represents the raw data, the right panel represents scale-free within-person centred data. It can be seen that the variable drug-free friends is virtually a constant and has no significant variance. As such, it can be excluded from statistical analysis.

*Satisfaction with life* was assessed using the Satisfaction With Life Scale (SWLS) sum score (30). The SWLS has demonstrated high validity and reliability (31). It is a self-reporting questionnaire which includes five items measuring the global life satisfaction experienced by the respondent. The SWLS has previously been used in research on this sample (21). See **Figure 1** for a graphic display of the trajectories of the four variables.

### 2.3 Data analysis

#### 2.3.1 Missing data

There are three main challenges with regard to missing data in the data set: a) participants missed whole measurement occasions, i.e. the intra-individual cluster size differs; b) participants provided incomplete data at a specific measurement occasion; c) a systematic missingness due to the design, as the time intervals from 1 – 24 months are three months, whereas the intervals from 24 – 60 months are 12 months.

**Table 2** gives an overview of cluster sizes. Of the 164 clusters (ids), an overwhelming 75% provided data at all eleven measurement occasions, and roughly 10% at ten. The smallest cluster size is 5 occasions, i.e. data were provided at about every other occasion, however, the percentage is very low. We can thus conclude that the data quality is very high with regard to problem a), i.e. cluster size. The intra-class correlations indicate a need for a hierarchical approach to data analysis (cf. **Table 3**).

**Table 1:** Baseline characteristics of included participants.

Variable	Specifier	Total (N=146)
Gender	Male	69 (62.2%)
	Female	42 (37.8%)
Age at baseline		27.1 (7.1)
Debut age substance use		13.1 (2.3)
Education level (completed)	Lower secondary school	65 (58.6%)
	Upper secondary school	31 (27.9%)
	Tertiary vocational education	14 (12.6%)
	Bachelor or higher	1 (0.9%)
Born in Norway		104 (93.7%)
Preferred drug^a^	Alcohol	5 (5%)
	Cannabis	21 (21.0%)
	Stimulants	19 (19.0%)
	Opioids	7 (7.0%)
	Benzodiazepines/sedatives	2 (2.0%)
	Multiple	24 (24.0%)
	Unknown	22 (22%)
History of intravenous drug use	No	47 (42.3%)
	Yes	64 (57.7%)
SCL-90-R GSI raw score at baseline		1.2 (0.7)
SWLS score at baseline		15.9 (6.2)

Data are presented as mean (SD) for continuous measures, and n (%) for categorical measures. ^a^All patients in this cohort used multiple substances, and this item refers to drug preference and not typical drug use. SCL-90-R GSI, Symptoms Checklist 90 Revised - Global Severity Index; SWLS, Satisfaction with life scale.

**Table 2:** Cluster size.

Cluster size (number of observations)	Percentage of total
5	1.8%
6	2.4%
7	1.2%
8	6.1%
9	3.7%
10	9.8%
11	75.0%

**Table 3:** Intra-class correlations.

Variable	ICC1
DUDIT	0.29
SWLS	0.45
SCL-90-R	0.49

Missing data analysis of the observed data, i.e. problem b), reveals that of all 164 × 11 = 1804 observations, 786 are complete. There are 220 incidents of missing data in all three variables at a given time point, 353 occasions with missing data in SCL-90-R, 389 occasions with missing in SCL-90-R and DUDIT, especially between 15 and 21 months but only 24 in SWLS and DUDIT. There are 651 occasions with missingness in DUDIT over all patterns. Dependence-analysis reveals that the data distribution of DUDIT does not depend on missingness in SCL-90-R, but clearly on missingness in SWLS. See **Supplementary Figures 1–3** in the **Supplementary Material** for a detailed depiction of the missingness patterns and dependencies. Given the relatively high number of complete data sets, the complex data-structure, and the observation that time-trends can hardly be modelled with imputation techniques, we chose not to impute missing data, especially since the DSEM is rather robust to missing data (32). However, in order to account for the unequally spaced time intervals, i.e. problem c), we included missing time points and coded the outcome as missing, i.e., generated a continuous time variable but did not impute data. Appending the data this way allows the autoregressive term to maintain a constant interpretation without interfering with within-level dependencies (33, 34).

#### 2.3.2 Dynamic structural equation model

To account for the complex data structure, with eleven observations of the three remaining variables nested in k=164 individuals, we fitted a Bayesian two-level dynamic structural equation model (DSEM). This approach allows us to analyse the within-person and the between-persons dynamics over time controlled for possible time sensitive and time-invariant confounders. Thus, the individuals’ dynamic changes were analysed by latent centring of the dependent variables and creating time-lagged (t-1) variables as predictors (20). However, since we assumed a trend in the data, i.e. considered the outcomes also as a function of time, we detrended the autoregression analysis and used the time-lag of the residuals rather than the variable itself (34). This approach allows for analysing the extent to which a preceding measurement occasion influences subsequent measurement occasions at the concurrent time while accounting for the trends in the data. The approach is beneficial to longitudinal SUD research as it becomes possible to assess the degree to which a variable affects substance use over time from one measurement occasion to the next while keeping an individual baseline for each participant. Since 11 measurement occasions are not sufficient to estimate random autoregressive (AR) slopes, we modelled the AR effects as fixed effects. The time trends, however, could be modelled as latent variables on the between-persons level. The variable ‘drug-free friends’ is binary-coded and appears to be relatively constant over time. It did not affect the other variables in the analyses and is therefore not included in the final model and is not reported in the results section. Lastly, we added age and gender as between-level predictors.

The model was fitted in Mplus (vs. 8.6, Muthen & Muthen). In order to ease convergence, the dependent variables were downscaled to similar ranges, i.e. DUDIT by factor 5 and SWLS by factor 10 (35). See **Supplementary Material**, section 2 for rescaling information. We first fitted the model with non-informative priors (reference model) to test convergence. In a second step, we included weakly informative admissible-range priors (36) to concentrate the probability mass on the relevant parameter space, which gives more accurate parameter estimates, especially for the variance terms and R^2^ values. Prior-sensitivity analysis revealed that the admissible range priors create more conservative results and shift the estimates towards the zero. We interpret this as an increment in precision. See **Supplementary Table 4** in the supplement for more information.

**Figure 2:**
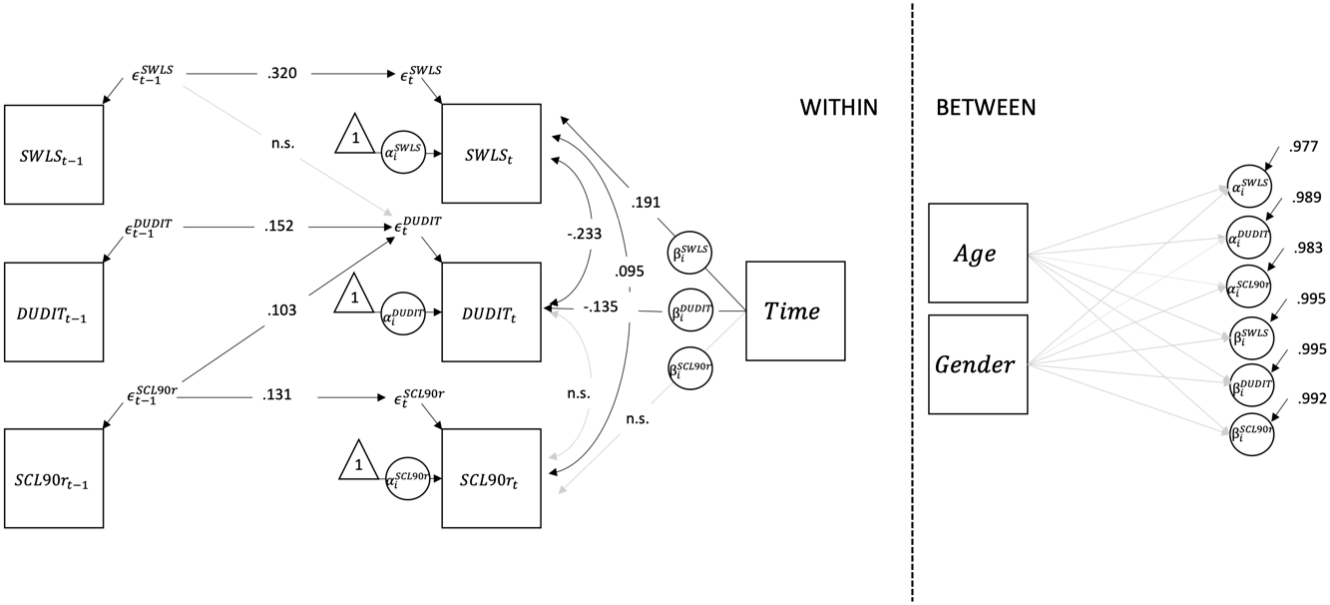
Structural model with standardized path coefficients. To detrend the time-series, the autoregressive terms were modelled on the residuals ∈ rather than on the variables themselves. The means and variances of the exogenous variables are not shown to focus on parameters of interest in the model. SCL-90-R, Symptoms Checklist 90 Revised; DUDIT, Drug Use Disorders Identification Test; SWLS, Satisfaction With Life Scale.

The model is displayed in **Figure 2**, Mplus-code and prior information are given in the **Supplementary Material**.

Model quality was determined using effective sample size (ESS), which estimates the amount by which autocorrelation in samples increases uncertainty relative to an independent sample (37); and the potential scale reduction factor Rhat, which tests for non-stationarity with a simulation chain by comparing the distributions of each chain (38). See **Supplementary Material**, section 2.7 for more information.

## 3 Results

### 3.1 Substance use

Fluctuations in the individuals’ means of substance use at the concurrent time point t are credibly predicted by fluctuations in the individuals’ means of psychological distress at t-1 and satisfaction with life at t-1 (est_std_ = 0.152, se_std_ = 0.061; see **Table 4** for details). Moreover, these fluctuations have a credible linear time effect (averaged over all observations: *TREND_1_* = -0.135, sd_std_ = 0.030), which translates to approximately one point decrease on the DUDIT scale per year (see **Supplementary Material** for rescaling algorithm), indicating a slight decline over time. However, these results did not change markedly when controlling for age and gender. The model explains 23.4% of the variance on the observation level, but only 1.1% on the between level for substance use (see **Table 5** for details).

**Table 4:** STDYX standardized results.

	Estimate	SD	lo95 CRI	up95 CRI	Sig.
Fixed time trend effects (within level)					
*TREND_1_|DUDIT_t_*	**-0.135**	**0.030**	**-0.190**	**-0.074**	***
*TREND_2_|SWLS_t_*	**0.191**	**0.032**	**0.129**	**0.253**	***
*TREND_3_|SCL90R_t_*	-0.036	0.035	-0.104	0.033	n.s.
Autoregressive terms on the residuals (within level)					
$\in_{t}^{DUDIT}\leftarrow \in_{t-1}^{DUDIT}$	**0.152**	**0.061**	**0.028**	**0.267**	***
$\in_{t}^{DUDIT}\leftarrow \in_{t-1}^{SWLS}$	0.025	0.064	-0.104	0.146	n.s.
$\in_{t}^{DUDIT}\leftarrow \in_{t-1}^{SCL90r}$	**0.103**	**0.051**	**0.004**	**0.200**	***
$\in_{t}^{SWLS}\leftarrow \in_{t-1}^{SWLS}$	**0.320**	**0.063**	**0.194**	**0.436**	***
$\in_{t}^{SCL90r}\leftarrow \in_{t-1}^{SCL90r}$	**0.131**	**0.044**	**0.039**	**0.212**	***
Covariances (within level)					
$DUDIT_{t}↔SWLS_{t}$	**-0.233**	**0.035**	**-0.299**	**-0.163**	***
$DUDIT_{t}↔SCL90r_{t}$	-0.038	0.045	-0.125	0.050	n.s
$SWLS_{t}↔SCL90r_{t}$	0.095	0.043	0.014	0.183	n.s.
Residual variances (within level)					
$DUDIT_{t}$	**0.766**	**0.020**	**0.726**	**0.805**	***
$SWLS_{t}$	**0.738**	**0.017**	**0.701**	**0.769**	***
$SCL90r_{t}$	**0.702**	**0.030**	**0.643**	**0.759**	***
Regressions (between level)					
$\beta_{i}^{DUDIT}\;\leftarrow Gender$	-0.012	0.060	-0.130	0.105	n.s.
$\beta_{i}^{DUDIT}\;\leftarrow Age$	-0.026	0.059	-0.141	0.089	n.s.
$\beta_{i}^{SWLS}\leftarrow Gender$	0.012	0.060	-0.107	0.127	n.s.
$\beta_{i}^{SWLS}\;\leftarrow Age$	0.001	0.059	-0.116	0.114	n.s.
$\beta_{i}^{SCL90r}\;\leftarrow Gender$	0.061	0.060	-0.060	0.175	n.s.
$\beta_{i}^{SCL90r}\leftarrow Age$	-0.001	0.059	-0.117	0.116	n.s.
$\alpha_{i}^{DUDIT}\;\leftarrow Gender$	-0.028	0.085	-0.195	0.138	n.s.
$\alpha_{i}^{DUDIT}\;\leftarrow Age$	-0.041	0.085	-0.209	0.124	n.s.
$\alpha_{i}^{SCL90r}\leftarrow Gender$	0.032	0.075	-0.111	0.180	n.s.
$\alpha_{i}^{SCL90r}\leftarrow Age$	-0.127	0.075	-0.270	0.023	n.s.
$\alpha_{i}^{SWLS}\leftarrow Gender$	0.016	0.073	-0.130	0.156	n.s.
$\alpha_{i}^{SWLS}\leftarrow Age$	-0.104	0.072	-0.246	0.036	n.s.
Covariances (between level)					
$DUDIT_{t}↔SWLS_{t}$	**-0.432**	**0.153**	**-0.724**	**-0.126**	***
$DUDIT_{t}↔SCL90r_{t}$	0.201	0.148	-0.088	0.491	n.s.
$SWLS_{t}↔SCL90r_{t}$	**-0.590**	**0.121**	**-0.826**	**-0.349**	***
Intercepts (between level)					
$\alpha_{i}^{DUDIT}$	**2.196**	**0.440**	**1.352**	**3.069**	***
$\alpha_{i}^{SCL90r}$	**1.985**	**0.354**	**1.299**	**2.685**	***
$\alpha_{i}^{SWLS}$	**4.001**	**0.535**	**3.028**	**5.109**	***
*TREND_1_|DUDIT_t_*	-0.069	0.255	-0.578	0.418	n.s.
*TREND_2_|SWLS_t_*	0.108	0.255	-0.396	0.599	n.s.
*TREND_3_|SCL90R_t_*	-0.264	0.253	-0.748	0.239	n.s.
Variances (between level)					
$\sigma_{i}^{TREND1}$	**0.995**	**0.008**	**0.976**	**1.000**	***
$\sigma_{i}^{TREND2}$	**0.995**	**0.007**	**0.978**	**1.000**	***
$\sigma_{i}^{TREND3}$	**0.992**	**0.010**	**0.969**	**1.000**	***
$\sigma_{i}^{DUDIT}$	**0.989**	**0.017**	**0.949**	**1.000**	***
$\sigma_{i}^{SWLS}$	**0.983**	**0.018**	**0.943**	**1.000**	***
$\sigma_{i}^{SCL90r}$	**0.977**	**0.022**	**0.929**	**1.000**	***

*** = significant, n.s.= not significant. Bold = significant.

**Table 5:** R^2^ values.

Variable	Estimate	SD	lo95 CRI	up95 CRI
Within level				
*DUDIT*	0.234	0.020	0.195	0.274
*SCL90R*	0.262	0.017	0.231	0.299
*SWLS*	0.298	0.030	0.241	0.357
Between Level				
*DUDIT*	0.011	0.017	0.000	0.051
*SCL90R*	0.017	0.018	0.000	0.057
*SWLS*	0.023	0.022	0.000	0.071
*TREND1*	0.005	0.008	0.000	0.024
*TREND2*	0.005	0.007	0.000	0.022
*TREND3*	0.008	0.010	0.000	0.031

### 3.2 Psychological distress

The fluctuation in the individuals’ means of psychological distress at t is positively predicted by deviations from the individuals’ means of psychological distress at t-1 (see **Table 3**). Moreover, higher psychological distress at t-1 credibly predicts slightly higher substance use at t (est_std_ = 0.103, sd_std_ = 0.051). We found no indications of statistically credible time-effect. The R^2^ for psychological stress is 0.262 on the observational level, and 0.023 on the between-level.

### 3.3 Satisfaction with life

The deviation in the individuals’ means of satisfaction with life at t is positively predicted by the deviation in the individuals’ means of psychological distress at t-1 (est_std_ = 0.320, sd_std_ = 0.063, see **Table 4** for details). Moreover, satisfaction with life increases over time (averaged over all observations: *TREND_2_* = 0.191, sd_std_ = 0.032, which translates to approximately three units on the original SWLS-scale within one year (see **Supplementary Material** for rescaling algorithm)). At the concurrent time point t, satisfaction with life is negatively correlated with substance use and psychological distress. The R^2^ for satisfaction with life is 0.298 on the observational level. The model further explains 2.3% of the variance between the individuals.

### 3.4 Drug-free friends

The drug-free friends variable was removed from the final model. It did not have any effect on the other variables (see discussion below for details). Our rational for not including this variable was to create a more parsimonious model.

## 4 Discussion

### 4.1 Substance use and psychological distress

Our findings suggest that higher-than-average psychological distress at a three-month lag credibly predicts higher-than-average drug use at the concurrent time point t, suggesting that experiencing higher psychological distress at the previous measurement occasion leads to higher substance use at the concurrent time point. This is in line with previous multi-level dynamic structural equation model indicating an association between substance use and lagged negative affect (39). Furthermore, previous research suggests that reduced substance use is associated with lowered psychological distress over three years (12) and that psychological distress may decline across several years following abstinence (13, 40). Consequently, it may be possible that reduced psychological distress facilitates abstinence or a reduction in substance use. However, our findings indicate that there is a time trend in substance use, but not in psychological distress. This suggests that these trajectories are asynchronous and that it might therefore be difficult to assess their long-term correlation. We expected to find an association between drug-free friends and psychological distress as previous research suggests a link between perceived social support and perceived stress (41), but our analyses found none.

### 4.2 Change takes time

Our findings show that fluctuations in the individuals’ means of satisfaction with life at both t and a three-month lag are negatively associated with the deviations in the individuals’ means of substance use at t. This suggests a synchronous pattern between satisfaction with life and substance use in the sense that higher satisfaction with life predicts lower substance use. However, as we did not analyse the directionality, this relationship could be inversed.

Our results suggest that a decrease in substance use after treatment is associated with increased satisfaction with life. This suggests that substance use reduction may improve satisfaction with life and *vice versa* and that substance use reduction may be a necessary part of obtaining and maintaining recovery. Our results indicate that substance use decreases over time while satisfaction with life increases, notably with small but significant individual differences.

Even though there is a possibility that some substance use may not deteriorate recovery (42) and that abstinence may not be necessary to achieve recovery (43, 44) our findings suggest that higher satisfaction with life is associated with lower substance use while higher psychological distress predicts higher substance use. This seems to suggest that abstinence or a reduction in substance use may be associated with higher satisfaction of life and reduced psychological distress.

### 4.3 Recovery is cumbersome and non-linear

Our results resonate with empirical findings stating that recovery is a cumbersome and non-linear process (26, 45–48). We found positive and negative fluctuations from one measurement occasion to the next across follow-ups over five years. Individuals’ mean deviations from psychological distress at a three-month lag positively indicate higher satisfaction with life and are credibly associated at the concurrent time point. Our results may be best explained by the stability of psychological distress over time in combination with strong fluctuations in satisfaction with life around the individuals’ means, indicating a rather asynchronous pattern. This suggests that recovery is not a linear process. However, to understand the association between these two variables, closer monitoring is warranted.

As seen in **Table 4**, psychological distress and satisfaction with life are, as expected, negatively associated at the between-persons level (non-centred). Nevertheless, the individuals’ deviations from their means are positively correlated: When individuals are above their means in psychological distress (i.e. are more distressed), they are also likely to be above their means in satisfaction with life. While this was unexpected, our findings may not be so curious given that recovery is a stressful and dynamic change process (3). Presumably, individuals in recovery oscillate between creating a new and drug-free life as community citizens while leaving their old life behind (26). Recovery is a challenging development process moving from a larger degree of dependence on others to being more independent (45, 46, 49, 50). This change process may impact satisfaction with life and psychological distress, thus contributing to positive and negative fluctuations. In this regard, the conflict between the “old” and the “new” life may increase psychological distress while at the same time increasing satisfaction with life as one struggles to adapt, while simultaneously finding the process satisfying.

### 4.4 Research implications

Our findings suggest negative and positive fluctuations in satisfaction with life, psychological distress, and substance use over five years. This indicates that SUD research may benefit from conducting several follow-ups of these measures with a more detailed temporal resolution to account for possible fluctuations.

### 4.5 Clinical implications

Patients and service providers’ knowledge of negative and positive fluctuations in satisfaction with life, substance use, and psychological distress may be useful for providing tailored and time-specific care. This supports the perspective that although relapse or lapse may happen, this can be considered as a part of the recovery process and not a sign of treatment failure. Knowing that well-being fluctuates over time may ease patients’ hearts in difficult periods. This may contribute to maintained motivation and increased perseverance. This is crucial as satisfaction with life is a key motivator and predictor of successful SUD treatment (15). Previous research suggests that functional solutions require the involvement of treatment professionals and positive social networks (41, 51, 52), and that these solutions would ideally be based on the service user’s existing skill set and wishes (53). A recent review has shown that several studies have reported an association between negative affect and craving in a bidirectional manner (54). This is in line with our findings indicate that negative periods are part of recovery. A possible clinical implication of this is that treatment interventions should aim to provide easy access to care under such circumstances, such as a planned in-patient stay, adaptive sequential treatment schedules, higher frequency of psychotherapy sessions, or other helpful measures based on the individual’s preferences.

### 4.6 Strengths and limitations

We consider it a strength that this study is one of the few SUD recovery studies with a time scope extending two years, focusing on social and psychological variables. While Dyar and colleagues have taken a similar Bayesian multilevel SEM approach in their analyses of minority stress, coping motives, and substance use among sexual minority women and gender-diverse individuals (55, 56), applying rDSEM in analysing addictive behaviours is, to our knowledge, novel and extremely beneficial: it allows accounting for time trends in autoregressive models while it does not require stationary data. This is especially interesting for SUD *recovery* research where one primary objective is to investigate positive treatment effects over time in relation to obtaining and maintaining recovery.

As in most longitudinal research, missing data limit the analytical possibilities and results; and this study is no exception. However, we tried our best to meticulously analyse and describe the problems and apply effective measures to mitigate falsified parameter estimates. The variable ‘drug-free friends’ is dichotomous and may not be sensitive enough to detect its interdependence with the other variables in our study. Furthermore, this variable may neither distinguish between drug-free friends with a history of substance use nor casual and problem substance use. A possible weakness is that we did not control for patients’ previous treatment experience in our analyses, as research have previously found these parameters to be associated with recovery.

## 5 Conclusion

Our study suggests that reduction in substance use is related to increase in satisfaction with life over five years. There were monthly and yearly positive and negative fluctuations in substance use and satisfaction with life indicating that recovery is a non-linear process. Psychological distress seems to be related to increased substance use and may be a risk factor for patient relapse. Our study found drug-free friends as conceived in this study not to impact the other variables probably due to its coarseness since previous research have found this variable to impact SUD patients’ recovery positively.

## Data availability statement

The raw data supporting the conclusions of this article will be made available by the authors, without undue reservation.

## Ethics statement

The studies involving humans were approved by the Regional Committees for Medical and Health Research Ethics (REK, 2011/1877), Norway. The studies were conducted in accordance with the local legislation and institutional requirements. In Norway, young people (aged between 16 and 18 years of age) consent on their own behalf when asked to participate in non-interventional medical and health research. Therefore, written informed consent for participation was not required from the participants’ legal guardians/next of kin in accordance with the national legislation and institutional requirements (ACT 2008-06-20, paragraph 17).

## Author contributions

FM: Conceptualization, Formal analysis, Project administration, Writing – original draft, Writing – review & editing. AE: Resources, Writing – original draft, Writing – review & editing. JB: Writing – original draft, Writing – review & editing. UD: Data curation, Formal analysis, Methodology, Supervision, Writing – original draft, Writing – review & editing.
