# Supplementary material for: The interdependence of substance use, satisfaction with life, and psychological distress: a dynamic structural equation model analysis
Source: Front Psychiatry. 2024 Feb 9;15:1288551. doi: 10.3389/fpsyt.2024.1288551 (PMC10884273; doi:10.3389/fpsyt.2024.1288551)
Supplement: Supplementary file 1 [file Presentation_1.pdf]

The interdependence of substance use, satisfaction with life, and psychological distress. A dynamic structural equation model analysis

Supplementary Material

Fredrik Moe, Aleksander Erga, Jone Bjørnestad, Ulrich Dettweiler

December 2023

Contents

1 Supplementary Results **2**
1.1 Missing Data Analysis 2
1.2 Rescaling of Estimates 2

2 Supplementary Information on the Statistical Approach **2**
2.1 Bayes' Rule 2
2.2 Prior Probability Functions 3
2.3 Likelihood Function 4
2.4 Prior Sensitiviy Analysis 8
2.5 Posterior Distribution 8
2.6 Bayesian Model Evaluation and Selection 8
2.7 Convergence and Autocorrelation Diagnosis 10

3 Computer-Code **11**
3.1 Determining Admissible Range Priors 11
3.2 Test priors in model with intercepts, variances, and covariances only 18
3.3 The rDSEM Model 19
3.4 R-code for further diagnoses 21

1 Supplementary Results

1.1 Missing Data Analysis

Panel A) in Figure S1 indicates that DUDIT-scores show the same distribution pattern irrespective of missingness in SCL90r. On the contrary, Panel C) shows that missingness of SWLS is highly correlated with missingness in DUDIT: DUDIT-values are missing mostly together with SWLS-values (missing = TRUE) and the distribution pattern of DUDIT-values is clearly different for missing=TRUE for SWLS from when they are not. Panel B) displays the overall missing pattern. To keep those dependencies in data imputation while also maintaining the intra-individual (within-cluster) dynamics is almost impossible; and given the overall very good quality of the data with relatively little missingness, imputation was not indicated.

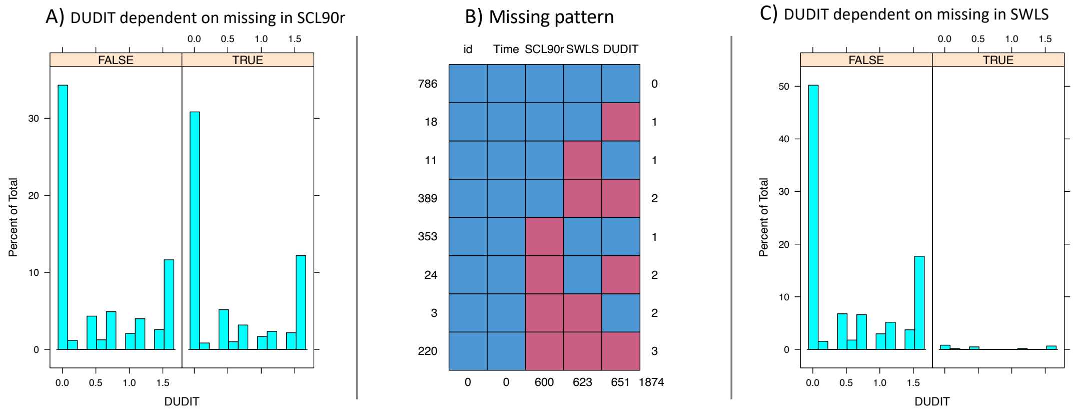

Figure S1: Missing Patterns

1.2 Rescaling of Estimates

DUDIT was down-scaled by factor 5. Variance=0.425, sd=0.652; Time: variance=32.661, sd=5.715. One unit (sd) up in time (+5.715 months) will lead to 5 times 0.652 times -0.135 units in DUDIT = -0.440.

SWLS was down-scaled by factor 10. Variance=0.475, sd=0.689. One unit (sd) up in time (+5.715 months) will lead to 10 times 0.689 times 0.191 units in SWLS = 1.316.

Table ´1 provides an overview of the number of observatinos per variable at the respective measurement occasions.

2 Supplementary Information on the Statistical Approach

2.1 Bayes' Rule

The Bayesian statistical approach evaluates the probability p of a hypotheses H, expressed by the parameters θ in a statistical model, directly by updating our prior beliefs about the probability of

Table 1: Number of observations per variable per measurement occasion

Month	DUDIT	SCL-90-R	SWLS
1	146	109	164
3	130	112	135
6	102	120	104
12	145	133	143
15	64	164	61
18	60	144	61
21	37	117	38
24	133	146	134
36	117	65	120
48	112	60	112
60	107	34	109

this hypothesis or the parameters, $p(\theta)$, given the data D and expressed by x in a statistical model, $p(H|D)$, by means of a likelihood function. Each of the three terms *posterior*, *likelihood*, and *prior* are *probability distributions* (pdfs).

- The *posterior* is the probability of the parameters given the data, $p(\theta|x)$,
- The *likelihood* is the probability of observing the data given the parameters (unknowns), $p(x|\theta)$,
- The *prior* represents external knowledge about the parameters, $p(\theta)$.

More formally expressed, the *posterior* is proportional to *likelihood* times *prior*, or

$$\text{posterior} \propto \text{likelihood} \times \text{prior}. \tag{1}$$

This is very different from the frequentist approach, which evaluates the probability of the data given a (never true) null-hypothesis, $p(D|H_0)$. As a general statement, we can state Baye's rule as follows

$$p(\theta|x) \propto p(x|\theta) \times p(\theta) \tag{2}$$

or, more fully:

$$p(\theta|x) = \frac{p(x|\theta) \times p(\theta)}{p(x)}. \tag{3}$$

2.2 Prior Probability Functions

In the Bayesian context, $P(\theta)$ is defined for each parameter in a so-called prior-distribution function which we have to specify in the models. This is the "subjective" part of the theorem. However, this "prior belief" must be justified; if no reliable information is available that helps us to formulate fully informed priors, we have several options: diffuse or flat priors, i.e. assigning the same probability to all possible values of θ, which comes closest to what frequentist statistics (i.e., maximum likelihood) does, which in a Bayesian work flow, can serve as a reference model [5]; weakly informed or vague priors, which limit the probability space for the parameters within a reasonable range; so-called "objective" priors, which put a mix of generic prior functions on the parameters; or hyper-priors, which

are more data-driven. For dynamic structural equation models (DSEM), McNeish has suggested to specify weakly informative admissible-range-restricted priors, which can be even done in the absence of previous studies ([8]). In rDSEM, where the autoregressions of the time-lags are estimated on the residuals, precise residual and variance terms are essential. As McNeish has shown, uninformative priors lead to biased results; we thus calculated the total variance and intraclass correlation coefficient (ICC) for each of the dependent three variables and derived the centrality metric and admissible range for each parameter from this information by centering the prior probability mass around the respective measure of central tendency. Variance parameters have been conceptualized as inverse gamma distributions (Γ^{-1}), using the mode as centrality measure; hereby, we tested different combinations of shape and rate parameters of inverse gamma distribution while keeping the mode constant. Intercepts and slope parameters were conceived as normal distributions, using the mean to define centrality. When appropriate, priors for classes of parameters have been vectorized with a reasonably wide distribution. See table 2 and figure S2 for specific information. R-code is provided in chapter 3 for the individual steps in the derivation.

2.3 Likelihood Function

The likelihood function determines the probability of observing the data given the parameters and represents the data-generating process in the simulation. Dependent on the data-structure of the variable of interest, the likelihood function can be chosen from a wide range of probability functions. Table 2 gives an overview over the probability functions used in this analysis.

Table 2: Distributions for Likelihood and Prior Probability Functions

Distribution	Range	Useful for
Normal, $N(\mu, \sigma^2)$	$(-\infty, \infty)$	Normally distributed data. A good default choice. Centrality is defined by the mean μ, dispersion by the variance σ^2 (in Mplus) or standard deviation sd or σ (in R).
Inverse Gamma, $IG(\alpha, \beta)$	$(0, \infty)$	Continuous data with a lower bound of zero, with shape parameter a and scale parameter b. The probability mass is left-skewed, leaning towards zero, with thick tails to the right, making this distribution very interesting for defining priors for variance terms. The mode of $\Gamma^{-1} = \frac{b}{a+1}$.

Table 3: Prior probability functions for the parameters and effective sample size estimates

Parameters	Prior	Mean	Variance	Std. Dev.	ESS	ESS/N
1, WITHIN: DUDIT ON DUDIT^{t-1}	N(0, 9)	0.00	9.00	3.00	2395	0.16
2, WITHIN: DUDIT ON SCL90R^{t-1}	N(0, 9)	0.00	9.00	3.00	3431	0.23
3, WITHIN: DUDIT ON SWLS^{t-1}	N(0, 9)	0.00	9.00	3.00	2141	0.14
4, WITHIN: SCL90R ON SCL90R^{t-1}	N(0, 9)	0.00	9.00	3.00	4489	0.30
5, WITHIN: SWLS ON SWLS^{t-1}	N(0, 9)	0.00	9.00	3.00	1874	0.12
6, WITHIN: DUDIT	IG(5, 2.1)	0.53	0.10	0.30	6679	0.45
7, WITHIN: SCL90R WITH DUDIT	N(0, 1)	0.00	1.00	1.00	4333	0.29
8, WITHIN: SCL90R	IG(5, 2.1)	0.53	0.10	0.30	5823	0.39
9, WITHIN: SWLS WITH DUDIT	N(0, 1)	0.00	1.00	1.00	6864	0.46
10, WITHIN: SWLS WITH SCL90R	N(0, 1)	0.00	1.00	1.00	4172	0.28
11, WITHIN: SWLS	IG(5, 2.1)	0.53	0.10	0.30	6866	0.46
12, BETWEEN: [TREND1]	N(0, 9)	0.00	9.00	3.00	6149	0.41
13, BETWEEN: [TREND2]	N(0, 9)	0.00	9.00	3.00	7092	0.47
14, BETWEEN: [TREND3]	N(0, 9)	0.00	9.00	3.00	3211	0.21
15, BETWEEN: [DUDIT]	N(0, 9)	0	9.00	3.00	5394	0.36
16, BETWEEN: [SCL90R]	N(0, 9)	0	9.00	3.00	4244	0.28
17, BETWEEN: [SWLS]	N(0, 9)	0	9.00	3.00	6898	0.46
18, BETWEEN: TREND1 ON GENDER	N(0, 9)	0.00	9.00	3.00	6535	0.44
19, BETWEEN: TREND1 ON AGE	N(0, 9)	0.00	9.00	3.00	6432	0.43
20, BETWEEN: TREND2 ON GENDER	N(0, 9)	0.00	9.00	3.00	7737	0.52
21, BETWEEN: TREND2 ON AGE	N(0, 9)	0.00	9.00	3.00	6660	0.44
22, BETWEEN: TREND3 ON GENDER	N(0, 9)	0.00	9.00	3.00	3156	0.21
23, BETWEEN: TREND3 ON AGE	N(0, 9)	0.00	9.00	3.00	3464	0.23
24, BETWEEN: DUDIT ON GENDER	N(0, 9)	0.00	9.00	3.00	5607	0.37
25, BETWEEN: DUDIT ON AGE	N(0, 9)	0.00	9.00	3.00	5899	0.39
26, BETWEEN: SCL90R ON GENDER	N(0, 9)	0.00	9.00	3.00	4230	0.28
27, BETWEEN: SCL90R ON AGE	N(0, 9)	0.00	9.00	3.00	4329	0.29
28, BETWEEN: SWLS ON GENDER	N(0, 9)	0.00	9.00	3.00	7072	0.47
29, BETWEEN: SWLS ON AGE	N(0, 9)	0.00	9.00	3.00	7128	0.48
30, BETWEEN: TREND1	IG(3, 0.16)	0.08	0.006	0.08	10163	0.68
31, BETWEEN: TREND2	IG(3, 0.16)	0.08	0.006	0.08	10611	0.71
32, BETWEEN: TREND3	IG(3, 0.16)	0.08	0.006	0.08	6507	0.43
33, BETWEEN: DUDIT	IG(5, 0.42)	0.11	0.004	0.061	2962	0.20
34, BETWEEN: SCL90R WITH DUDIT	N(0, 1)	0.00	1.00	1.00	3365	0.22
35, BETWEEN: SCL90R	IG(5, 0.66)	0.17	0.009	0.095	3885	0.26
36, BETWEEN: SWLS WITH DUDIT	N(0, 1)	0.00	1.00	1.00	2603	0.17
37, BETWEEN: SWLS WITH SCL90R	N(0, 1)	0.00	1.00	1.00	4259	0.28
38, BETWEEN: SWLS	IG(5, 0.66)	0.17	0.009	0.095	2612	0.17

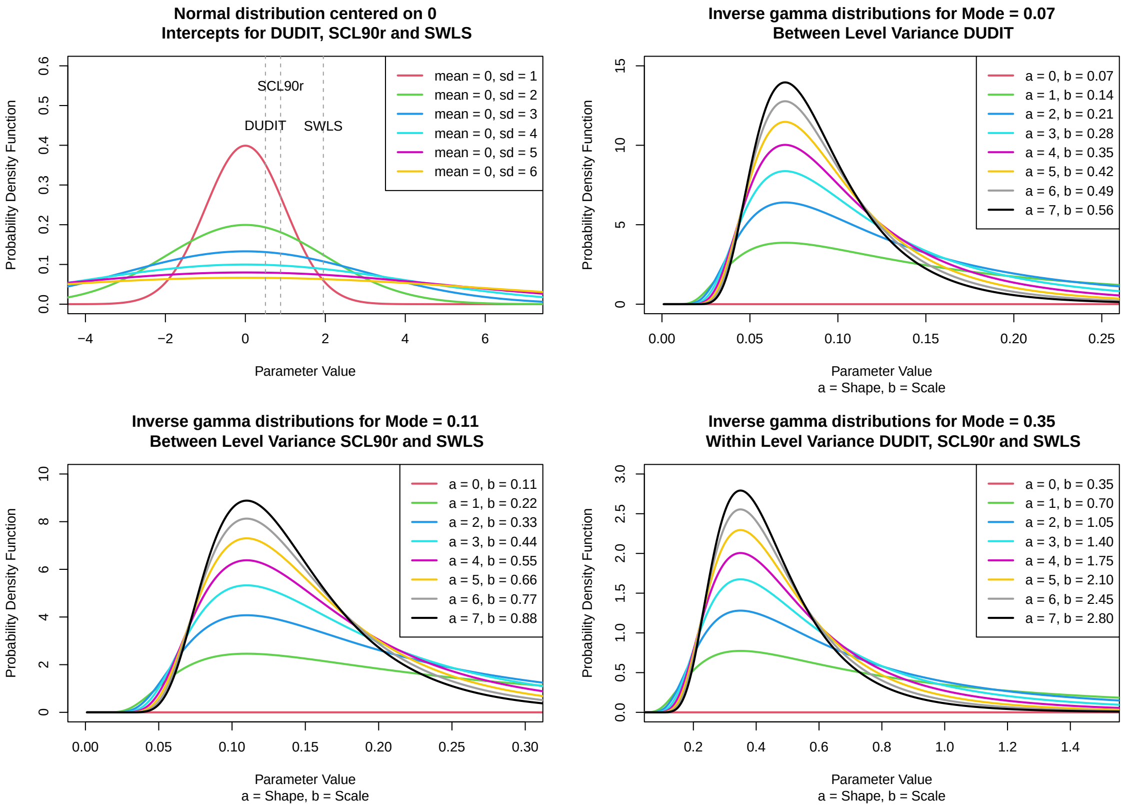

Figure S2: Admissible-range prior distributions used for the intercepts and variance terms (within and between levels).

Table 4: Admissible ranges for the respective priors

Mode	a = shape	b = scale	HDI lower	HDI upper
0.35	0	0.35	inf	inf
0.35	1	0.7	0.065	13.653
0.35	2	1.05	0.100	2.965
0.35	3	1.4	0.123	1.725
0.35	4	1.75	0.140	1.295
0.35	5	2.1	0.154	1.081
0.35	6	2.45	0.165	0.953
0.35	7	2.8	0.174	0.868
0.35	8	3.15	0.182	0.807
0.35	9	3.5	0.189	0.761

Mode	a = shape	b = scale	HDI lower	HDI upper
0.11	0	0.11	inf	inf
0.11	1	0.22	0.020	4.291
0.11	2	0.33	0.031	0.932
0.11	3	0.44	0.039	0.542
0.11	4	0.55	0.044	0.407
0.11	5	0.66	0.048	0.340
0.11	6	0.77	0.052	0.300
0.11	7	0.88	0.055	0.273
0.11	8	0.99	0.057	0.254
0.11	9	1.1	0.060	0.241

Between level variance DUDIT, mode = 0.07

Mode	a = shape	b = scale	HDI lower	HDI upper
0.07	0	0.07	inf	inf
0.07	1	0.14	0.013	2.731
0.07	2	0.21	0.020	0.593
0.07	3	0.28	0.028	0.259
0.07	4	0.35	0.028	0.259
0.07	5	0.42	0.031	0.216
0.07	6	0.49	0.033	0.191
0.07	7	0.56	0.035	0.174
0.07	8	0.63	0.036	0.161
0.07	9	0.7	0.038	0.152

Intercept for DUDIT, SCL90r and SWLS, mean = 0

mean	sd	HDI lower	HDI upper
0	1	-1.960	1.960
0	2	-3.920	3.920
0	3	-5.880	5.880
0	4	-7.840	7.840
0	5	-9.800	9.800
0	6	-11.760	11.760

2.4 Prior Sensitiviy Analysis

Table 5 presents the increase in precision from the reference model with flat priors to the admissible range priors using the unstandandardized poterior parameter extimates. It can be seen that the admissible range priors create more conservative results and shift the estimates towards the zero. We interpret this as an increase in precision and robustness.

2.5 Posterior Distribution

By applying Bayes' rule, a posterior probability distribution for each parameter in the model is then estimated by means of the likelihood function times the priors(s). Since Bayes rule can only be directly applied to very simple models, posterior distributions of more complex models are estimated via Markov-Chain Monte Carlo (MCMC) simulation whose algorithms allow to approximate multidimensional integrals. Markov chain Monte Carlo methods create samples from continuous random variables, whose probability densities are proportional to the prior probability function. These samples can be used to evaluate an integral over that variable, as its expected value or variance. To run those simulations, several independent chains of draws are developed, starting from a set of points arbitrarily (or more mindfully) chosen and sufficiently distant from each other. These chains are stochastic processes of "walkers" which move around randomly according to an algorithm that looks for places with a reasonably high contribution to the integral to move into next, assigning them higher probabilities. In complex models, this process leads to three problems: high uncertainty (non-convergence) of the draws within a chain, non-convergence between the chains, and highly autocorrelated draws. To mitigate convergence problems, a reasonably high number of draws ("iterations", "runs") need to be performed and the algorithm needs to be given some time to stabilise, a process named "burnin". By default, Mplus discards the first half of the chains. To further facilitate convergence, transforming the variable values to similar scales and ranges (e.g. z-standardizing) helps to find good starting values for the algorithm, which then can work from there to find values with highest probabilities. Autocorrelation can be reduced by "thinning" the chain, i.e. taking only e.g. every 3rd or 5th draw. The actual number of draws used in a simulation is thus:

$$N = \frac{\text{number of chains} \times \text{iterations}}{\text{thinning}} \times \text{proportion of burnin}. \tag{4}$$

As the Bayesian inference returns a distribution of possible effect values (the posterior), the quantiles of the credibility interval (CRI) can directly be inspected [10]. CRIs define the range containing a particular percentage of probable values and summarize the uncertainty related to the estimated parameters. With symmetrical likelihood functions (e.g. $N(\mu, \sigma^2)$, or $U(a, b)$), the CRI can be expressed with en equally tailed interval (ETI). For instance, the 95% CRI is simply the central portion of the posterior distribution that contains 95% of the values, with 2.5% outside the interval at either side of the curve given by the likelihood function. In this analysis, all likelihood functions were symmetrical.

2.6 Bayesian Model Evaluation and Selection

To evaluate how observed data are represented in the MCMC-simulation, Bayesian analysts use posterior predictive checks (PPC). The estimated model is considered to be true if the data in the simulation are generated according to the likelihood function. In other words, the unobserved parameters are generated by the prior probability functions, and then, using parameter draws in the

Table 5: Prior Sensitivty Analysis

Parameter	Estimate		Precicion [%]	sd		Precicion [%]
Priors	flat	admissible range		flat	admissible range	
1	0.141	0.152	7.2	0.059	0.06	1.7
2	-0.011	0.024	145.8	0.064	0.068	5.9
3	0.052	0.121	57.0	0.058	0.06	3.3
4	0.338	0.318	6.3	0.053	0.066	19.7
5	0.156	0.133	17.3	0.04	0.044	9.1
6	-0.067	-0.058	15.5	0.009	0.009	0.0
7	-0.02	-0.01	100.0	0.011	0.011	0.0
8	0.028	0.021	33.3	0.009	0.01	10.0
9	0.289	0.281	2.8	0.014	0.014	0.0
10	0.212	0.211	0.5	0.01	0.01	0.0
11	0.227	0.225	0.9	0.011	0.011	0.0
12	-0.002	-0.002	0.0	0.002	0.009	77.8
13	0	-0.002	100.0	0.001	0.004	75.0
14	0.002	0.002	0.0	0.002	0.009	77.8
15	0	0	.n.d.	0.001	0.004	75.0
16	0.005	0.009	44.4	0.003	0.009	66.7
17	0	0	n.d.	0.001	0.004	75.0
18	-0.034	-0.026	30.8	0.083	0.08	3.8
19	-0.023	-0.018	27.8	0.039	0.038	2.6
20	0.036	0.04	10.0	0.095	0.093	2.2
21	-0.068	-0.075	9.3	0.045	0.044	2.3
22	0.066	0.02	230.0	0.091	0.092	1.1
23	-0.068	-0.062	9.7	0.043	0.043	0.0
24	-0.066	-0.056	17.9	0.018	0.022	18.2
25	0.06	0.026	130.8	0.018	0.02	10.0
26	-0.137	-0.101	35.6	0.023	0.024	4.2
27	0.692	0.69	0.3	0.11	0.114	3.5
28	0.761	0.838	9.2	0.131	0.131	0.0
29	1.681	1.681	0.0	0.135	0.133	1.5
30	-0.002	-0.003	33.3	0.003	0.013	76.9
31	0.005	0.005	0.0	0.003	0.012	75.0
32	-0.006	-0.013	53.8	0.004	0.012	66.7
33	0.116	0.098	18.4	0.021	0.024	12.5
34	0.191	0.175	9.1	0.028	0.031	9.7
35	0.183	0.172	6.4	0.029	0.036	19.4
36	0	0.002	100.0	0	0	n.d.
37	0	0.002	100.0	0	0	n.d.
38	0	0.002	100.0	0	0	n.d.

MCMC-simuation, the observed data are generated by the likelihood. The relation of the estimated model and the observed data is often expressed by the Posterior Predictive P-value (PPP). A PPP of 0.5 means that a test statistic $T(y)$ will be exactly equal to the median of the posterior predictive distribution of $T(y_{rep})$. If a model's predictions are "biased" to be too high, then we will get a number greater than 0.5, and if they are generally on the low side, we will get a number less than 0.5. A $PPP > 0.05$ is considered a minimal threshold for model fit [9].

In DSEM, however, observations are not independent across time as the models are fitting autoregressions. Thus, posterior predictive checking is not available at the current state of the art. Tihomir Asparouhov recommends to estimate models as unrestricted as the data allow and then walk them back to obtain a more parsimonious model by removing insignificant effects (link to Mplus discussion site).
We thus fitted a series of nested models, with and without random slopes for the autoregressive terms, with and without the time-invariant ("between") control variables age and gender, as well as with and without drug-free friends as time-sensitive ("within") predictor and autoregressive outcome. None of the latter variables had significant impact on the parameter estimates of interest, however, including age and gender as time-invariant covariates explained more variance and was theoretically preferred, despite a higher deviance information criterion (DIC): DIC with age and gender: 20520.3, effective number of parameters: 6983.6; DIC without: 17775.3, effective number of parameters: 5786.7. The DIC is a hierarchical modeling generalization of the Akaike information criterion (AIC). It is particularly useful in Bayesian model selection problems where the posterior distributions of the models have been obtained by Markov chain Monte Carlo (MCMC) simulation. As with the AIC, relatively lower DIC is better.

2.7 Convergence and Autocorrelation Diagnosis

To evaluate convergence, we used the potential scale reduction factor (PRSF, also represented as $\hat{R}$), which belongs to the family of chain-convergence diagnostics tools. In equilibrium, the distribution of samples from chains should be the same regardless of the initial starting values of the chains [2]. One way to check this is to compare the distributions of multiple chains—in equilibrium they should all have the same mean. Additionally, the split $\hat{R}$ tests for convergence by splitting the chain in half and testing the hypothesis that the means are the same in each half, given by a ratio of the two parts of $mean_1/mean_2 = 1$. This tests for non-stationarity within a chain. All parameters must show convergence. This is a necessary but not sufficient condition for convergence [1]. The rule of thumb is that $\hat{R}$ values for all parameters are less than 1.05.

Autocorrelation was checked with the proportion of the effective sample size ESS to the actual sample size N in MCMC-simulations, ESS/N, which indicates how much autocorrelation in the MCMC sampling reduced the actual number of independent draws. The effective sample size (ESS) measures the amount by which autocorrelation in samples, expressed as the autocorrelation function $ACF(k)$, increases uncertainty (standard errors) relative to an independent sample. According to Kass et al. [7], the ESS can approximated by the following equation:

$$ESS = \frac{N}{1 + 2 \times \sum_{k=1}^{\infty} ACF(k)}. \tag{5}$$

We considered the models to perform adequately with $\hat{R} < 1.05$ [3] and $ESS/N > 0.1$ [4] [6].

We ran the model with five independent chains, each containing 30.000 iterations, where the first half is discarded as burn in. The thinning rate was set to 5, thus, given equation (4), $N =$

$\frac{5\times30000}{5} \times 2^{-1} = 15000$ iterations. The model converged well after 01:47:53 hours using MPlus 8 (1.8.8) on a MacBook Pro M1 running on Ventura 13.0 with all $\hat{R} \leq 1.01$.
ESS/N can be approximated by rearranging equation (5), since $ACF(k)$ can be extracted from Mplus via the .gh5-object and further processed in R (see code below). Hereby, it is necessary to cut-off the sum over the autocorrelations at a value of k where the autocorrelations seem to have fallen to near zero, as including estimates for lots of higher lags adds too much noice [7]. As a convention, this should be the case at lag $k = 20$. The formula to be used is then:

$$ESS/N = \frac{1}{1 + 2 \times \sum_{k=1}^{20} ACF(k)}. \tag{6}$$

Effective sample size and autocorrelations are acceptable (see Table 3).

3 Computer-Code

3.1 Determining Admissible Range Priors

```
# Calculate variances and ICC
library(lme4)
library(dplyr)

# DUDIT
m <- lmer(DUDIT ~ 1 + (1 | USERNAME), data = data)
summary(m)
out_m <- as.data.frame(VarCorr(m))

between_var_m <- out_m[1,4]
residual_var_m <- out_m[2,4]
total_var_m <- between_var_m + residual_var_m
ICC_ID_m <- between_var_m/total_var_m

# Extract intercept
dd <- ranef(m)[["USERNAME"]]
dd <- cbind(Subject=rownames(dd),dd)
rownames(dd) <- NULL
m_tibble <- dplyr::add_rownames(coef(m)[["USERNAME"]])
m.df <- as.data.frame(m_tibble)
i_DUDIT <- mean(m.df$`(Intercept)`)

# SCL90r
n <- lmer(SCL90r ~ 1 + (1 | USERNAME), data = data)
summary(n)
out_n <- as.data.frame(VarCorr(n))

between_var_n <- out_n[1,4]
residual_var_n <- out_n[2,4]
total_var_n <- between_var_n + residual_var_n
ICC_class_n <- between_var_n/total_var_n
```

```
ee <- ranef(n)[["USERNAME"]]
ee <- cbind(Subject=rownames(ee),ee)
rownames(ee) <- NULL
n_tibble <- dplyr::add_rownames(coef(n)[["USERNAME"]])
n.df <- as.data.frame(n_tibble)
i_SCL90r <- mean(n.df$`(Intercept)`)

# SWLS
o <- lmer(SWLS ~ 1 + (1 | USERNAME), data = data)
summary(o)
out_o <- as.data.frame(VarCorr(o))

between_var_o <- out_o[1,4]
residual_var_o <- out_o[2,4]
total_var_o <- between_var_o + residual_var_o
ICC_class_o <- between_var_o/total_var_o

ff <- ranef(o)[["USERNAME"]]
ff <- cbind(Subject=rownames(ff),ff)
rownames(ff) <- NULL
o_tibble <- dplyr::add_rownames(coef(o)[["USERNAME"]])
o.df <- as.data.frame(o_tibble)
i_SWLS <- mean(o.df$`(Intercept)`)

# Reasonable mode of between variance prior SWLS, SCL90r:
mno_b <- c(between_var_n, between_var_o)
Med_Var_b = round(mean(mno_b),2) # 0.11

# Reasonable mode of within variance prior:
mno_w <- c(residual_var_m, residual_var_n, residual_var_o)
Med_Var_w = round(mean(mno_w),2) # 0.35




# Calcualte addissible range for priors
library(invgamma)
library(HDInterval)

##### Intercept ####

int <- function(x)
    qnorm(x,0,sd=1)
round(hdi(int),3) # -1.959964  1.959964
int <- function(x)
    qnorm(x,0,sd=2)
round(hdi(int),3) # -3.919928  3.919928
int <- function(x)
    qnorm(x,0,sd=3)
hdi(int) # -5.879892  5.879892
int <- function(x)
    qnorm(x,0,sd=4)
```

```
hdi(int) # -7.839856  7.839856
int <- function(x)
    qnorm(x,0,sd=5)
round(hdi(int),3) # -9.79982  9.79982
int <- function(x)
    qnorm(x,0,sd=6)
round(hdi(int),3) # -11.75978  11.75978




int <- function(x)
  qinvgamma(x,shape=2,rate=0.12)
hdi(int)

######## Between level variance DUDIT, Mode = 0.07 #########

int <- function(x)
  qinvgamma(x,shape=0,rate=0.07)
hdi(int)
# inf Inf
int <- function(x)
  qinvgamma(x,shape=1,rate=0.14)
hdi(int)
# 0.01303464 2.73061467, mode = 0.07
int <- function(x)
  qinvgamma(x,shape=2,rate=0.21)
hdi(int)
# 0.01994004 0.59301111

int <- function(x)
  qinvgamma(x,shape=3,rate=0.28)
hdi(int)
# 0.02809585 0.25899803

int <- function(x)
  qinvgamma(x,shape=4,rate=0.35)
hdi(int)
# 0.02809585 0.25899803

int <- function(x)
  qinvgamma(x,shape=5,rate=0.42)
hdi(int)
# 0.03080738 0.21618686

int <- function(x)
  qinvgamma(x,shape=6,rate=0.49)
hdi(int)
# 0.03300554 0.19062813

int <- function(x)
  qinvgamma(x,shape=7,rate=0.56)
```

```
hdi(int)
# 0.03483698 0.17361717

int <- function(x)
  qinvgamma(x,shape=8,rate=0.63)
hdi(int)
# 0.03639545 0.16144852

int <- function(x)
  qinvgamma(x,shape=9,rate=0.70)
hdi(int)
# 0.0391916 0.1953193

########  Within Level Variance DUDIT, SCL90R and SWLS, mode = 0.35 #########

int <- function(x)
  qinvgamma(x,shape=0,rate=0.35)
hdi(int)
# inf Inf

int <- function(x)
  qinvgamma(x,shape=1,rate=0.70)
hdi(int)
# 0.06517321 13.65307335

int <- function(x)
  qinvgamma(x,shape=2,rate=1.05)
hdi(int)
# 0.0997002 2.9650556

int <- function(x)
  qinvgamma(x,shape=3,rate=1.40)
hdi(int)
# 0.1231258 1.7248863

int <- function(x)
  qinvgamma(x,shape=4,rate=1.75)
hdi(int)
# 0.1404793 1.2949901

int <- function(x)
  qinvgamma(x,shape=5,rate=2.10)
hdi(int)
# 0.1540369 1.0809343

int <- function(x)
  qinvgamma(x,shape=6,rate=2.45)
hdi(int)
# 0.1650277 0.9531407

int <- function(x)
  qinvgamma(x,shape=7,rate=2.80)
```

```
hdi(int)
# 0.1741849 0.8680858

int <- function(x)
  qinvgamma(x,shape=8,rate=3.15)
hdi(int)
# 0.1819773 0.8072426

int <- function(x)
  qinvgamma(x,shape=9,rate=3.5)
hdi(int)
# 0.1887205 0.7614330


######## Between Level Variance SCL90R and SWLS, mode = 0.11 ######

int <- function(x)
  qinvgamma(x,shape=0,rate=0.11)
hdi(int)
# inf Inf

int <- function(x)
  qinvgamma(x,shape=1,rate=0.22)
hdi(int)
# 0.02048301 4.29096591

int <- function(x)
  qinvgamma(x,shape=2,rate=0.33)
hdi(int)
# 0.03133435 0.93187460

int <- function(x)
  qinvgamma(x,shape=3,rate=0.44)
hdi(int)
# 0.03869667 0.54210712

int <- function(x)
  qinvgamma(x,shape=4,rate=0.55)
hdi(int)
# 0.04415063 0.40699690

int <- function(x)
  qinvgamma(x,shape=5,rate=0.66)
hdi(int)
# 0.0484116 0.3397222

int <- function(x)
  qinvgamma(x,shape=6,rate=0.77)
hdi(int)
# 0.05186584 0.29955849

int <- function(x)
```

```
  qinvgamma(x,shape=7,rate=0.88)
hdi(int)
# 0.05474382 0.27282698

int <- function(x)
  qinvgamma(x,shape=8,rate=0.99)
hdi(int)
# 0.05719285 0.25370482

int <- function(x)
  qinvgamma(x,shape=9,rate=1.11)
hdi(int)
# 0.05985134 0.24148304

# Plot the priors for better understanding
par(mfrow=c(2,2))
# Intercetps
t <- seq(-5, 8, .01)
plot(t, dnorm (t,0, sd=1), col = 2, lwd = 2, type = 'l',
     ylab = "Probability␣Density␣Function",
     ylim = c(0,0.6),
     xlim = c(-4,7),
     xlab = "Parameter␣Value",
     main = "Normal␣distribution␣centered␣on␣0
␣␣␣␣␣Intercepts␣for␣DUDIT,␣SCL90r␣and␣SWLS")
lines(t, dnorm(t,0,sd=2), col = 3, lwd = 2, type = 'l')
lines(t, dnorm(t,0,sd=3), col = 4, lwd = 2, type = 'l')
lines(t, dnorm(t,0,sd=4), col = 5, lwd = 2, type = 'l')
lines(t, dnorm(t,0,sd=5), col = 6, lwd = 2, type = 'l')
lines(t, dnorm(t,0,sd=6), col = 7, lwd = 2, type = 'l')

legend(x = "topright",          # Position
       legend = c("mean␣=␣0,␣sd␣=␣1",
                  "mean␣=␣0,␣sd␣=␣2",
                  "mean␣=␣0,␣sd␣=␣3",
                  "mean␣=␣0,␣sd␣=␣4",
                  "mean␣=␣0,␣sd␣=␣5",
                  "mean␣=␣0,␣sd␣=␣6"), # Legend texts

       lty = c(1,1,1,1,1,1),       # Line types
       col = c(2,3,4,5,6,7),       # Line colors
       lwd = 2)                      # Line width
abline(v=c(i_DUDIT, i_SWLS, i_SCL90r),
       col= (c(8,8,8)),
       lty = c(2,2,2))
text(c(i_DUDIT, i_SWLS, i_SCL90r), c(0.45, 0.45, 0.55), c("DUDIT", "SWLS", "SCL90r"))

s <- seq(0, 3, .001)
plot(s, dinvgamma (s,0, 0.07), col = 2, lwd = 2, type = 'l',
     ylab = "Probability␣Density␣Function",
     ylim = c(0,15),
     xlim = c(0,0.25),
```

```
     xlab = "Parameter Value",
     main = "Inverse gamma distributions for Mode = 0.07
     Between Level Variance DUDIT",
     sub = "a = Shape, b = Scale")
lines(s, dinvgamma(s,1,0.14), col = 3, lwd = 2, type = 'l')
lines(s, dinvgamma(s,2,0.21), col = 4, lwd = 2, type = 'l')
lines(s, dinvgamma(s,3,0.28), col = 5, lwd = 2, type = 'l')
lines(s, dinvgamma(s,4,0.35), col = 6, lwd = 2, type = 'l')
lines(s, dinvgamma(s,5,0.42), col = 7, lwd = 2, type = 'l')
lines(s, dinvgamma(s,6,0.49), col = 8, lwd = 2, type = 'l')
lines(s, dinvgamma(s,7,0.56), col = 9, lwd = 2, type = 'l')
legend(x = "topright",           # Position
       legend = c("a = 0, b = 0.07",
                  "a = 1, b = 0.14",
                  "a = 2, b = 0.21",
                  "a = 3, b = 0.28",
                  "a = 4, b = 0.35",
                  "a = 5, b = 0.42",
                  "a = 6, b = 0.49",
                  "a = 7, b = 0.56"),  # Legend texts
       lty = c(1,1,1,1,1,1,1,1),       # Line types
       col = c(2,3,4,5,6,7,8,9),       # Line colors
       lwd = 2)                      # Line width


# Between Level Variance SCL90R and SWLS, mode = 0.11

plot(s, dinvgamma (s,0, 0.11), col = 2, lwd = 2, type = 'l',
     ylab = "Probability Density Function",
     ylim = c(0,10),
     xlim = c(0,0.30),
     xlab = "Parameter Value",
     main = "Inverse gamma distributions for Mode = 0.11
     Between Level Variance SCL90r and SWLS",
     sub = "a = Shape, b = Scale")
lines(s, dinvgamma(s,1,0.22), col = 3, lwd = 2, type = 'l')
lines(s, dinvgamma(s,2,0.33), col = 4, lwd = 2, type = 'l')
lines(s, dinvgamma(s,3,0.44), col = 5, lwd = 2, type = 'l')
lines(s, dinvgamma(s,4,0.55), col = 6, lwd = 2, type = 'l')
lines(s, dinvgamma(s,5,0.66), col = 7, lwd = 2, type = 'l')
lines(s, dinvgamma(s,6,0.77), col = 8, lwd = 2, type = 'l')
lines(s, dinvgamma(s,7,0.88), col = 9, lwd = 2, type = 'l')
legend(x = "topright",           # Position
       legend = c("a = 0, b = 0.11",
                  "a = 1, b = 0.22",
                  "a = 2, b = 0.33",
                  "a = 3, b = 0.44",
                  "a = 4, b = 0.55",
                  "a = 5, b = 0.66",
                  "a = 6, b = 0.77",
                  "a = 7, b = 0.88"),  # Legend texts
       lty = c(1,1,1,1,1,1,1,1),       # Line types
```

```
       col = c(2,3,4,5,6,7,8,9),       # Line colors
       lwd = 2)                       # Line width

# Within Level Variance DUDIT, SCL90R and SWLS, mode = 0.35

plot(s, dinvgamma (s,0, 0.35), col = 2, lwd = 2, type = 'l',
     ylab = "Probability␣Density␣Function",
     ylim = c(0,3),
     xlim = c(0.1,1.5),
     xlab = "Parameter␣Value",
     main = "Inverse␣gamma␣distributions␣for␣Mode␣=␣0.35
␣␣␣␣␣Within␣Level␣Variance␣DUDIT,␣SCL90r␣and␣SWLS",
     sub = "a␣=␣Shape,␣b␣=␣Scale")
lines(s, dinvgamma(s,1,0.70), col = 3, lwd = 2, type = 'l')
lines(s, dinvgamma(s,2,1.05), col = 4, lwd = 2, type = 'l')
lines(s, dinvgamma(s,3,1.40), col = 5, lwd = 2, type = 'l')
lines(s, dinvgamma(s,4,1.75), col = 6, lwd = 2, type = 'l')
lines(s, dinvgamma(s,5,2.10), col = 7, lwd = 2, type = 'l')
lines(s, dinvgamma(s,6,2.45), col = 8, lwd = 2, type = 'l')
lines(s, dinvgamma(s,7,2.80), col = 9, lwd = 2, type = 'l')
legend(x = "topright",          # Position
       legend = c("a␣=␣0,␣b␣=␣0.35",
                  "a␣=␣1,␣b␣=␣0.70",
                  "a␣=␣2,␣b␣=␣1.05",
                  "a␣=␣3,␣b␣=␣1.40",
                  "a␣=␣4,␣b␣=␣1.75",
                  "a␣=␣5,␣b␣=␣2.10",
                  "a␣=␣6,␣b␣=␣2.45",
                  "a␣=␣7,␣b␣=␣2.80"),  # Legend texts
       lty = c(1,1,1,1,1,1,1,1),       # Line types
       col = c(2,3,4,5,6,7,8,9),       # Line colors
       lwd = 2)                       # Line width
```

3.2 Test priors in model with intercepts, variances, and covariances only

Model code for the "null-model":

```
TITLE: Model to test the  "Admissable␣range␣restricted␣priors" (McNeish 2019)
DATA: FILE = "data.dat";

VARIABLE:

      VARIABLE:
      NAMES = USERNAME id TimeNew NAF SCL90r DUDIT SWLS Gender Age AgeZ TimeZ;
      USEVAR =  DUDIT SCL90r SWLS;
      MISSING=.;
      CLUSTER = USERNAME;


      ANALYSIS:   TYPE = TWOLEVEL RANDOM;
                  ESTIMATOR = BAYES;
                  FBITERATIONS=10000;
```

```
                PROC = 4;
                CHAINS = 3;
                THIN = 3;
                BSEED = 46956 ;

MODEL:
%WITHIN%

DUDIT SWLS SCL90r (e1-e3);

DUDIT WITH SWLS SCL90r (var1-var2);
SWLS WITH SCL90r (var3);

%BETWEEN%
[ DUDIT  SWLS SCL90r] (l2i1-l2i3);
  DUDIT  SWLS SCL90r (l2v1-l2v3);

DUDIT WITH SWLS SCL90r (l2var1-l2var2);
SWLS WITH SCL90r (l2var3)

MODEL PRIORS:

e1-e3 ~ IG(5, 2.10);
var1-var3 ~ N(0, 1);
l2i1-l2i3 ~ N(0, 9);
l2v1 ~ IG(5, 0.42);
l2v2-l2v3 ~ IG(5, 0.66);
l2var1-l2var3 ~ N(0, 1);

OUTPUT:
STAND TECH1 TECH4 TECH8 cinterval(hpd); ;
PLOT:
TYPE = PLOT2 PLOT3;
```

The model converged well with a PPP=0.516.

3.3 The rDSEM Model

MPlus code for final the Bayesian rDSEM we fitted is presented below. For the imputation of the missing time points with the TINTERVAL command, a time-variable needs to defined, if the time-trends should be run on the original time-scale. Thus, the autoregressive terms are modelled on the new time variable, while the time-trends are estimated with the original distances. The MODEL consists of two parts:

1. The "Within" part specifies:
   (a) the intra-person autoregressive relationships of the residuals, denoted with $\hat{t}$ for t and $\hat{1}$ for the time-lagged variable, including the cross-loadings;
   (b) the time trends for the variables;
   (c) the covariance terms;

    (d) the variances.

2. The "Between" part specifies:

    (a) the random intercepts, denoted in [...];

    (b) the variances of the random intercepts.

Moreover, the priors for the respective parameters are defined as moderately informative to concentrate the probability mass to a sensible range of data points.

```
TITLE: rDSEM with Age and Gender as between level variables and Admissible Range Priors
  DATA: FILE = "data.dat";

  VARIABLE:
  DEFINE:
      Time=TimeNew;
        VARIABLE:
        NAMES = USERNAME id TimeNew NAF SCL90r DUDIT SWLS Gender Age AgeZ TimeZ;
        TINTERVAL = TimeNew (3);
        USEVAR =  DUDIT SCL90r SWLS Gender AgeZ Time ;
        WITHIN = Time;
        BETWEEN = Gender AgeZ;
        LAGGED = DUDIT (1) SCL90r(1) SWLS(1);
        MISSING=.;
        CLUSTER = USERNAME;


        ANALYSIS:   TYPE = TWOLEVEL RANDOM;
                    ESTIMATOR = BAYES;
                    FBITERATIONS=25000;
                    PROC = 4;
                    CHAINS = 5;
                    THIN = 5;
                    BSEED = 46956 ;

      MODEL:
      %WITHIN%

      DUDIT^ ON DUDIT^1 (phi1);
      SWLS^ ON SWLS^1  (phi2); ! auto-regressive lag of resdiual
      SCL90r^ ON SCL90r^1 (phi3);

      DUDIT^ ON SWLS^1 (xi1); ! cross-lag for DUDIT
      DUDIT^ ON SCL90r^1 (xi2);

      Trend1 | DUDIT ON Time ;
      Trend2 | SWLS ON Time;
      Trend3 | SCL90r ON Time ;
      DUDIT SWLS SCL90r (e1-e3);

      DUDIT WITH SWLS SCL90r (var1-var2);
```

```
SWLS WITH SCL90r (var3);

%BETWEEN%
[ DUDIT  SWLS SCL90r Trend1 Trend2 Trend3] (l2i1-l2i6);
  DUDIT  SWLS SCL90r (l2v1-l2v3);
Trend1 Trend2 Trend3 (l2v4-l2v6);
  DUDIT  SWLS SCL90r Trend1 Trend2 Trend3 ON Gender AgeZ (l2s1-l2s12);
DUDIT WITH SWLS SCL90r (l2var1-l2var2);
SWLS WITH SCL90r (l2var3)

MODEL PRIORS:
phi1-phi3 ~ N(0,9); ! normal distribution, centered on 0, sd=3
xi1-xi2 ~ N(0,9);
!Trend1-Trend3 ~ N(0,25); No priors allowed
e1-e3 ~ IG(5, 2.10); ! Within-level variances for DUDIT, SWLS, SCL90r
var1-var3 ~ N(0, 1)); ! Covariances
l2i1-l2i3 ~ N(0, 9); ! Intercept for DUDIT, SWLS, SCL90r
l2i4-l2i6 ~ N(0, 9); ! Intercept for time trends
l2v1 ~ IG(5, 0.42); ! Between-level variance for DUDIT
l2v2-l2v3 ~ IG(5, 0.66); ! Between-level variance for SWLS and SCL90r
l2v4-l2v6 ~ IG(3, 0.16); ! Between-level variance for time trends, mode = 0.04
l2s1-l2s12 ~ N(0, 9);
l2var1-l2var3 ~ N(0, 1);

OUTPUT:
STAND TECH1 TECH4 TECH8 cinterval(hpd); ;
PLOT:
TYPE = PLOT2 PLOT3;
```

3.4 R-code for further diagnoses

R-code for missing analysis, calculation of ICC, and model fit evaluation:

```
# Missing data analysis
library(mice)
# Do DUDIT values depend on missing in SWLS? -> Yes, pattern different
histogram(~ DUDIT | is.na(SWLS), data=data)
# Do DUDIT values depend on missing in SCL90r? -> No, pattern pattern similar
histogram(~ DUDIT | is.na(SCL90r), data=data)

# Get the parameters from the gh5 Mplus output file
library(rhdf5)
mplus.list.bayesian.parameters('out.gh5')


# Store auto-correlations of all 23 parameters in data frame (from chain 2)
auto.corP_1 <- mplus.get.bayesian.autocorrelation('out.gh5',c(1:38),1)
auto.corP_2 <- mplus.get.bayesian.autocorrelation('out.gh5',c(1:38),2)
auto.corP_3 <- mplus.get.bayesian.autocorrelation('out.gh5',c(1:38),3)
auto.corP_4 <- mplus.get.bayesian.autocorrelation('out.gh5',c(1:38),4)
auto.corP_5 <- mplus.get.bayesian.autocorrelation('out.gh5',c(1:38),5)
```

```
autocorr.dat1 <- as.data.frame(auto.corP_1)
autocorr.dat2 <- as.data.frame(auto.corP_2)
autocorr.dat3 <- as.data.frame(auto.corP_3)
autocorr.dat4 <- as.data.frame(auto.corP_4)
autocorr.dat5 <- as.data.frame(auto.corP_5)


# Apply formula to calculate n_eff/N for the first 20 iterations
# of all parameters

N_eff_N1 <- with(autocorr.dat1, 1/(1+2*colSums(autocorr.dat1[1:20,])))
N_eff_N2 <- with(autocorr.dat2, 1/(1+2*colSums(autocorr.dat2[1:20,])))
N_eff_N3 <- with(autocorr.dat3, 1/(1+2*colSums(autocorr.dat3[1:20,])))
N_eff_N4 <- with(autocorr.dat4, 1/(1+2*colSums(autocorr.dat4[1:20,])))
N_eff_N5 <- with(autocorr.dat5, 1/(1+2*colSums(autocorr.dat5[1:20,])))

ESS_N <- round((N_eff_N1+N_eff_N2+N_eff_N3+N_eff_N4+N_eff_N5)/5,2)

# Print ESS / N
write.csv(ESS_N, file = "ESS_N.csv")

# Stack in one data frame
n_eff_Table_Chains <- as.data.frame(rbind(N_eff_N1, N_eff_N2,
                                    N_eff_N3, N_eff_N4, N_eff_N5))
n_eff_Table_Chains$Chain <- c("Chain␣1", "Chain␣2",
                              "Chain␣3", "Chain␣4", "Chain␣5")
N_eff_N_round2 <- round(n_eff_Table_Chains[,1:38], 2)

# Effective Sample Size
ESS_1 <- with(autocorr.dat1,15000/(1+2*colSums(autocorr.dat1[1:20,])))
ESS_2 <- with(autocorr.dat2, 15000/(1+2*colSums(autocorr.dat2[1:20,])))
ESS_3 <- with(autocorr.dat3,15000/(1+2*colSums(autocorr.dat3[1:20,])))
ESS_4 <- with(autocorr.dat4,15000/(1+2*colSums(autocorr.dat4[1:20,])))
ESS_5 <- with(autocorr.dat5,15000/(1+2*colSums(autocorr.dat5[1:20,])))

ESS <- round((ESS_1+ESS_2+ESS_3+ESS_4+ESS_5)/5,0)


# Print ESS
write.csv(ESS, file = "ESS.csv")

# Print autocorrelations for all parameters in all chains
write.csv(N_eff_N_round2[,1:38], file = "autocorrelations_num.csv")

# Check if n_eff/N >= 0.1 for all parameters in all chains
write.csv(ifelse(N_eff_N_round2[,1:38] >= 0.1, "OK", "CAUTION"),
file="autocorrelations_cat.csv")

# all n_eff/N >= 0.1
```

References

[1] Stephen P Brooks and Andrew Gelman. "General Methods for Monitoring Convergence of Iterative Simulations". In: *Journal of Computational and Graphical Statistics* 7.4 (1998), pp. 434–455. DOI: 10.1080/10618600.1998.10474787.

[2] Bob Carpenter et al. "Stan: A probabilistic programming language". In: *Journal of Statistical Software* 76.1 (2017). ISSN: 15487660. DOI: 10.18637/jss.v076.i01.

[3] Andrew Gelman and Donald B Rubin. "Inference from Iterative Simulation Using Multiple Sequences". In: *Statistical Science* 7.4 (1992), pp. 457–472. DOI: 10.1214/ss/1177011136. URL: https://doi.org/10.1214/ss/1177011136.

[4] Andrew Gelman et al. *Bayesian data analysis.* Third edit. Boca Raton, Florida: CRC Press, 2013, xiv, 667 pages. ISBN: 9781439840955.

[5] Andrew Gelman et al. "Bayesian workflow". In: *arXiv* (2020). ISSN: 23318422.

[6] Lei Gong and James M Flegal. "A Practical Sequential Stopping Rule for High-Dimensional Markov Chain Monte Carlo". In: *Journal of Computational and Graphical Statistics* 25.3 (2016), pp. 684–700. DOI: 10.1080/10618600.2015.1044092. URL: https://doi.org/10.1080/10618600.2015.1044092.

[7] Robert E. Kass et al. "Markov Chain Monte Carlo in Practice: A Roundtable Discussion". In: *The American Statistician* 52.2 (1998), pp. 93–100. (Visited on 12/19/2022).

[8] Daniel McNeish. "Two-Level Dynamic Structural Equation Models with Small Samples". In: *Structural Equation Modeling* 26.6 (Nov. 2019), pp. 948–966. ISSN: 15328007. DOI: 10.1080/10705511.2019.1578657.

[9] Bengt Muthén and Tihomir Asparouhov. "Bayesian structural equation modeling: A more flexible representation of substantive theory". In: *Psychological Methods* 17.3 (2012), pp. 313–335. DOI: 10.1037/a0026802.

[10] Rens van de Schoot et al. "A gentle introduction to bayesian analysis: applications to developmental research". In: *Child Dev* 85.3 (2014), pp. 842–860. DOI: 10.1111/cdev.12169. URL: https://www.ncbi.nlm.nih.gov/pubmed/24116396%20https://www.ncbi.nlm.nih.gov/pmc/articles/PMC4158865/pdf/cdev0085-0842.pdf.
